# Multi-locus phylogeny of dolphins in the subfamily Lissodelphininae: character synergy improves phylogenetic resolution

**DOI:** 10.1186/1471-2148-6-87

**Published:** 2006-11-01

**Authors:** April D Harlin-Cognato, Rodney L Honeycutt

**Affiliations:** 1 Department of Zoology, 203 Natural Sciences Building, Michigan State University, East Lansing, Michigan, 48823, USA; 2 Natural Science Division, Pepperdine University, 24255 Pacific Coast Highway, Malibu, California, 90263, USA

## Abstract

**Background:**

Dolphins of the genus *Lagenorhynchus *are anti-tropically distributed in temperate to cool waters. Phylogenetic analyses of cytochrome *b *sequences have suggested that the genus is polyphyletic; however, many relationships were poorly resolved. In this study, we present a combined-analysis phylogenetic hypothesis for *Lagenorhynchus *and members of the subfamily Lissodelphininae, which is derived from two nuclear and two mitochondrial data sets and the addition of 34 individuals representing 9 species. In addition, we characterize with parsimony and Bayesian analyses the phylogenetic utility and interaction of characters with statistical measures, including the utility of highly consistent (non-homoplasious) characters as a conservative measure of phylogenetic robustness. We also explore the effects of removing sources of character conflict on phylogenetic resolution.

**Results:**

Overall, our study provides strong support for the monophyly of the subfamily Lissodelphininae and the polyphyly of the genus *Lagenorhynchus*. In addition, the simultaneous parsimony analysis resolved and/or improved resolution for 12 nodes including: (1) *L. albirostris*, *L. acutus*; (2) *L. obscurus *and *L. obliquidens*; and (3) *L. cruciger *and *L. australis*. In addition, the Bayesian analysis supported the monophyly of the *Cephalorhynchus*, and resolved ambiguities regarding the relationship of *L. australis*/*L. cruciger *to other members of the genus *Lagenorhynchus*. The frequency of highly consistent characters varied among data partitions, but the rate of evolution was consistent within data partitions. Although the control region was the greatest source of character conflict, removal of this data partition impeded phylogenetic resolution.

**Conclusion:**

The simultaneous analysis approach produced a more robust phylogenetic hypothesis for *Lagenorhynchus *than previous studies, thus supporting a phylogenetic approach employing multiple data partitions that vary in overall rate of evolution. Even in cases where there was apparent conflict among characters, our data suggest a synergistic interaction in the simultaneous analysis, and speak against *a priori *exclusion of data because of potential conflicts, primarily because phylogenetic results can be less robust. For example, the removal of the control region, the putative source of character conflict, produced spurious results with inconsistencies among and within topologies from parsimony and Bayesian analyses.

## Background

**Figure 1:**
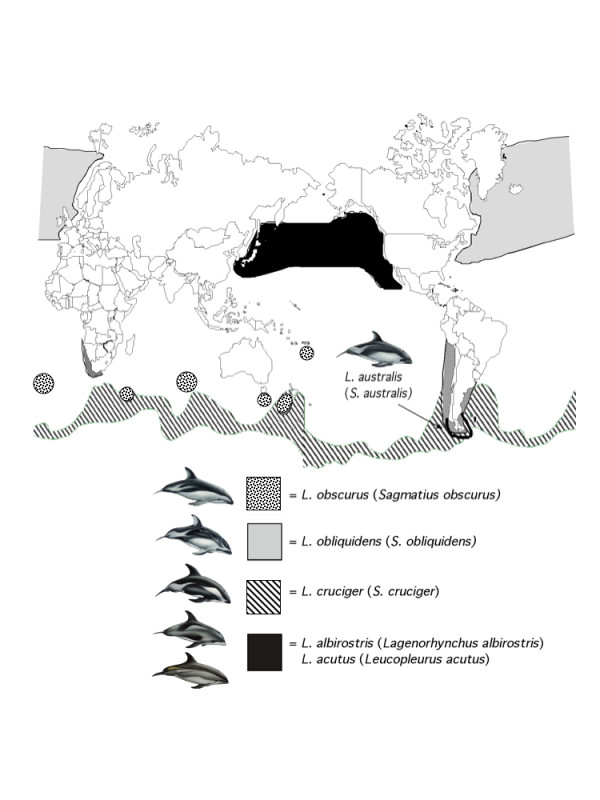
**Geographic distribution of the genus *Lagenorhynchus***. The subfamily Lissodelphininae (sensu LeDuc et al. [13]) includes all Southern Hemisphere species and *L. obliquidens *from the North Pacific. Generic names suggested by the taxonomic revision of Le Duc et al. [13] are indicated in parentheses, with *Lagenorhynchus *retained for the original type specimen (*L. albirostris*) first described by Gray [7].

Dolphins of the genus *Lagenorhynchus *are distributed in temperate to cool waters in the North Pacific, North Atlantic, and Southern oceans [1-4] (Figure 1). A short rostrum, relatively small, stout bodies, and flanks with horizontal flares of various contrasting patterns characterize all members of the genus. Within this group there is considerable variation in social structure and habitat, from coastal, shallow water Peale's dolphin (*L. australis*) that occurs in small groups within the Strait of Magellan and nearby fijords, to the meso-pelagic dusky dolphin (*L. obscurus*) that aggregates in groups of thousands along the continental shelves of New Zealand, South Africa, and South America.

Historically, color patterns, number of teeth, and the ratio of rostrum to brain case length were used as diagnostic characters to define dolphin genera, including the genus *Lagenorhynchus *[5-7]. However, these characters are now known to vary with sex and age within species [5]. It is not surprising, therefore, that the classification of dolphin groups based on these characters proved particularly problematic for early taxonomists. For *Lagenorhynchus*, the ubiquitous use of these morphological characters resulted at one time or another in specimens being assigned (and re-assigned) to at least 8 different genera (e.g., *Delphinus*, [7]; *Electra*, [8]; *Phocoena*, [9]; *Tursio*, [10]; *Leucopleurus*, [8]; *Clymenia*, [10]; *Sagmatius*, [11]). Recently, molecular systematic studies of mitochondrial cytochrome *b *(cyt *b*) and control region (dloop) sequences were employed to address the taxonomic ambiguities within the family Delphinidae. The results of these studies, based on a single representative from each species, suggested a polyphyletic *Lagenorhynchus*, leaving many other relationships weakly supported and the overall taxonomic status of the group unresolved [12,13]. For example, the cyt *b *cladogram of LeDuc et al. [13] indicated strong support for a monophyletic subfamily Lissodelphininae that contained *L. obliquidens, L. obscurus, L. australis*, *L. cruciger*, and the genera *Cephalorhynchus *and *Lissodelphis *but excluded the North Atlantic *L. albirostris *and *L. acutus*. A more recent Bayesian analysis of cyt *b *sequences by May-Collado and Agnarsson [14] supported the paraphyly of the genus *Lagenorhynchus *as suggested by LeDuc et al. [13], but increased taxonomic sampling (particularly of outgroups) provided increased phylogenetic resolution within the Lissodelphininae. Nevertheless, as with LeDuc et al. [13], some of the relationships among species of *Lagenorhynchus *and *Cephalorhynchus *within the Lissodelphininae remained unresolved.

Because of its rapid rate of coalescence and lack of recombination, mitochondrial DNA has been the molecule of choice for detailed studies of intra- and inter-specific evolution. However, the mitochondrial genome is a single, maternally inherited locus, and thus provides a perspective based on a single gene tree of female lineages. It is possible that the lack of phylogenetic resolution in LeDuc et al.'s [13] study was caused by limitations of a single, mitochondrial locus and/or the result of rapid divergence among taxa. However, distinguishing between alternative phylogenetic hypotheses requires the addition of more data from multiple loci and a detailed evaluation of the utility of various data partitions to resolve relationships within this group. To date, no further molecular systematic studies have attempted to address these issues.

In this paper we use four molecular markers (two nuclear and two mitochondrial) that vary in overall rates of evolution to diagnose relationships among species of dolphin in the genus *Lagenorhynchus *and among genera within the subfamily Lissodelphininae [13]. To increase taxonomic representation, we include 34 individuals representing 9 species of the Lissodelphininae and North Atlantic *Lagenorhynchus*. In addition, we utilize *a posteriori *measures to quantify the interaction among data partitions in a simultaneous analysis, and to characterize the effects of removing sources of conflict on phylogenetic resolution under the optimality criterion of parsimony and with the Bayesian method. Several studies have presented methods to quantify the interaction of characters in simultaneous analyses [15-17]. These measures are either: (1) topological indices that measure the change in either tree length or structure ("topological congruence") [18,19], and (2) measures of change in the amount of support at a particular node [15,17,20]. The reliability of these measures and their philosophical foundation has been questioned. In a recent review, Grant and Kluge ([21], pg. 409) advocated that *a posteriori *analyses of character partitions are heuristic only when "based on the results of the total-evidence analysis," and that there is "a great potential for the development of heuristic methods of *a posteriori *analysis of sets of characters." In this study, we attempt to heuristically examine with statistical analyses the contributions and interactions of data partitions in the resolution of relationships within the family Delphinidae. Our study is the first to use both nuclear and mitochondrial loci for the diagnosis of relationships within the family Delphinidae, and thus provides a unique opportunity to explore the utility of multiple process partitions in addressing the evolutionary history of this group.

## Results

### Simultaneous analysis and relationships within the Delphinidae

**Figure 2:**
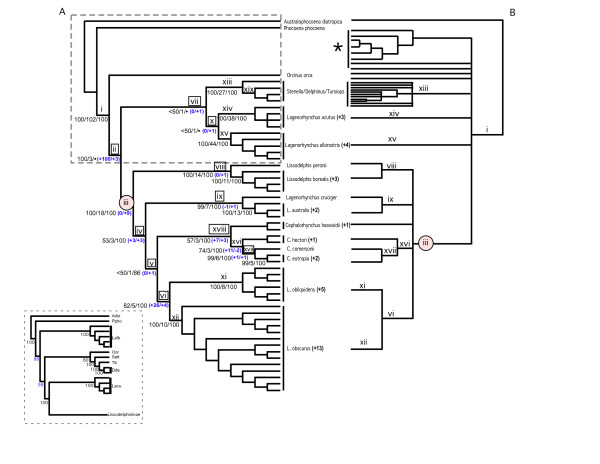
**A comparison of the simultaneous analysis tree derived from nuclear and mitochondrial genes to the single-locus cladogram of LeDuc et al. [13]**. **A)**. Strict consensus of 4 equally parsimonious trees (length = 1328, CI = 0.55, RI = 0.80) derived from the simultaneous analysis of Actin, RAG2, cytochrome *b*, and control region data. This tree is congruent with the results of the Bayesian analysis, except those nodes in the boxed insert (bottom left). Numbers to the left of each node are bootstrap proportions, decay indices, and posterior probabilities, respectively. Boxes mark nodes that have either improved resolution or are newly resolved in the simultaneous analysis; numbers in parentheses measure the change (increase, '+'; decrease, '-') in support indices. A circle denotes the Lissodelphininae clade. **B)**. The cytochrome *b *cladogram of LeDuc et al. [13]. Node numbers match those of the simultaneous analysis tree. The asterisk '*' indicates an additional clade of species represented in the LeDuc et al. [13] study but absent in our data set.

The simultaneous analysis of two nuclear and two mitochondrial genes (a total of 3,053 characters) produced four equally parsimonious trees that differed only in their level of intra-specific resolution (Figure 2). Fourteen of the 15 species-level clades had bootstrap values >99% and high decay indices (5 to 44) (Figure 2). Our results provide unambiguous support for the monophyly of the subfamily Lissodelphininae (node iii, Figure 2) and the polyphyly of the genus *Lagenorhynchus *(nodes x, ix, and vi, Figure 2), first suggested by LeDuc et al. [13] and May-Callado and Agnarsson [14]. Several relationships previously ambiguous in LeDuc et al. [13] were well-resolved in the simultaneous analysis (Figure 2) and included: (1) monophyly of *L. albirostris, L*. *acutus *and *Stenella*/*Tursiops*/*Delphinus *clades (nodes xv, xiv, and xiii, Figure 2); (2) increased support for the sister taxon relationship of *L. obscurus *and *L. obliquidens *(node vi); and (3) the monophyly *L. cruciger *and *L. australis *(node ix). Overall, the simultaneous parsimony analysis either resolved or improved resolution for 12 nodes (Figure 2). The Bayesian analysis produced a topology very similar to that of the parsimony analysis but with increased support at nodes that had low bootstrap values in the simultaneous parsimony analysis (e.g., nodes iv, v, xviii, Figure 2). Most notably, Bayesian analysis supported the monophyly of the *Cephalorhynchus *(node xviii, Figure 2), and resolved ambiguities in previous studies [13,14] regarding the relationship of *L. australis/L. cruciger *to other members of the genus *Lagenorhynchus*. There were some topological differences between the parsimony and Bayesian trees, but these were restricted to clades outside the Lissodelphininae where taxonomic sampling was relatively poor (Figure 2).

Separate analysis of data partitions produced topologies with variation in the degree of resolution (results not shown). For example, the cyt *b *partition produced a topology most similar to those of the simultaneous analysis, with 100% consensus among all inter-specific clades. Overall, the mitochondrial partitions had a greater number of phylogenetically informative characters and produced fewer most parsimonious trees than nuclear DNA data. Nevertheless, in all cases the simultaneous analysis cladogram provided considerably greater resolution than any of the independent analyses. In several cases, clades supported by the simultaneous analysis were also recovered in one or more of the independent analyses, with the exception of the control region – many of the relationships in this tree were not recovered in other analyses.

### Character dynamics: phylogenetic utility

**Figure 3:**
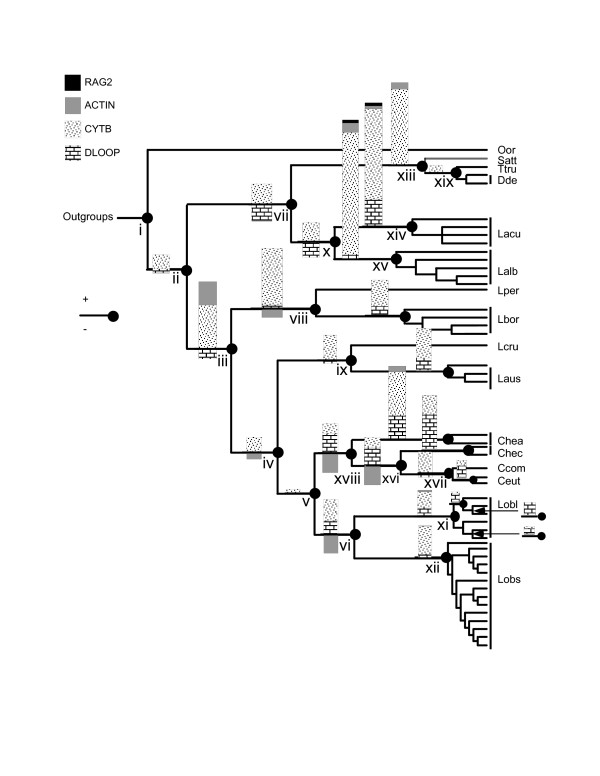
**A summary of the magnitude and distribution of partitioned branch support in the simultaneous analysis tree**. At each node, bars represent the relative partitioned branch support (PBS) contributed by a given data partition to a node. Shaded bars above the branch indicate positive PBS values; below the branch are negative PBS values. Nodes are numbered as in Figure 2.

**Figure 4:**
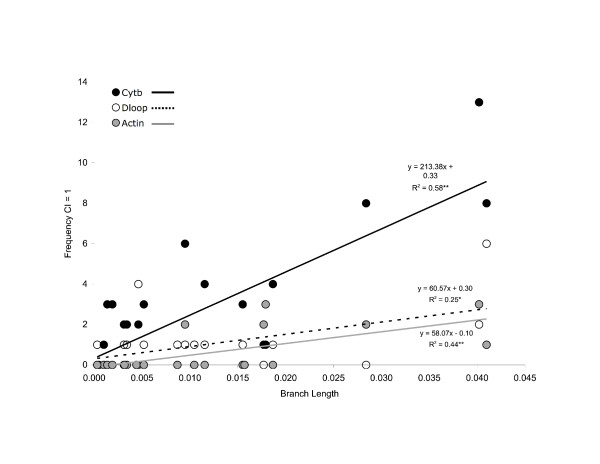
**Relationship between frequency of CI = 1 characters and branch lengths in the simultaneous analysis**. Results of linear regression analysis, *P ≤ 0.05, **P ≤ 0.01. Branch lengths were estimated as described in the text.

Each of the four process partitions contributed in some degree to the resolution of the simultaneous analysis tree (Table 2, Figure 3). On a node-by-node basis, the amount of localized topological support varied by dataset and among regions of the simultaneous analysis tree. For example, partitioned Bremer support values for cyt *b *were consistently positive at all nodes, while values for the control region were positive at the tips of the tree but negative at the base (Table 2, Figure 3). Longer branches had statistically more CI = 1 characters than shorter branches in the tree (H = 7.63, df = 2, *P *= 0.02), and in all data partitions there was a significant, positive relationship between the frequency of CI = 1 characters and branch length (cyt *b*, F = 29.5, df = 1, 21, *P *< 0.01; dloop F = 7.2, df = 1, 21, *P *< 0.05; Actin, F = 16.1, df = 1, 21, *P *< 0.01) (Figure 4). The strength of this relationship varied among data partitions, with cyt *b *having the steepest positive relationship between branch length and CI = 1 characters (Figure 4). Interestingly the dloop, although generally considered to evolve at a faster rate than cyt *b*, had fewer CI = 1 characters overall, and a slope more similar to that of Actin (Figure 4). Cyt *b *consistently provided the largest proportion of CI = 1 characters at each node (Table 2, Figure 4). Despite the greater frequency and wider distribution of highly consistent characters in cyt *b*, the relative proportion of support contributed by each data partition did not differ statistically among categories of branch length (χ^2 ^= 10.42, df = 6, *P *= 0.12), although Actin tended to contribute more to resolution along longer branches (Table 2). Put another way, each data partition consistently contributed the same *relative *proportion of the total number of CI = 1 characters among branch length categories, but the *absolute *number of these characters differed among partitions. Our results further suggest that 3rd position transitions occurred more frequently and provided more topological support than other classes of substitution (χ^2 ^= 12.4, df = 4, *P *= 0.02).

**Table 2:** Summary of support indices for branches in the simultaneous and independent analyses of data partitions.

	**Branch**	**Σ**	**Cytb**			**Dloop**			**Actin**			**RAG2**		
**Branch**	**length**	**CI1**	**CI1**	**HS**	**PB**	**CI1**	**HS**	**PB**	**CI1**	**HS**	**PB**	**CI1**	**HS**	**PB**

Out-i	0.0503	60	35	0	58.6	6	-1	17.4	15	0	23.0	3	0	3.0
i-ii	0.0046	6	2	0	4.9	4	-1	-0.8	0	-	-1.1	0	-	0
ii-iii	0.0179	5	1	0	14.0	1	-1	-2.5	3	-	-2.0	0	-	0
iii-iv	0.0034	3	2	1	1.0	1	0	0.7	0	-	0	0	-	0
iv-v	0.0008	0	0	-	1.0	0	-	0	0	-	0	0	-	0
v-vi	0.0003	0	0	1	6.6	1	-2	3.8	0	-	-5.4	0	-	0
vi-xi	0.0157	0	0	1	4.0	0	-1	3.0	0	-	1	0	-	0
vi-xii	0.0044	0	0	1	2.9	0	-1	1.0	0	-	-0.2	0	-	0
v-x	0.0105	1	0	1	3.1	1	1	5.9	0	-	-6.1	0	-	0
x-Chea	0.0115	5	4	0	14.0	1	0	7.0	0	-	2	0	-	0
x-xvi	0.0032	0	0	0	3.0	0	0	6.0	0	-	-6	0	-	0
xvi-Chec	0.0095	9	6	0	6.0	1	0	11.0	2	-	0	0	-	0
xvi-xvii	0.0014	3	3	0	7.0	0	0	-1.0	0	-	0	0	-	0
xvii-Ceut	0.0087	1	0	0	1.0	1	0	4.0	0	-	0	0	-	0
iv-ix	0.0177	3	1	0	8.0	0	-	-1.0	2	-	0	0	-	0
ix-Laus	0.0052	4	3	0	10.0	1	0	3.0	0	-	0	0	-	0
iii-viii	0.0187	5	4	1	18.0	1	-	-1.0	0	-	-3.0	0	-	0
viii-Lper	0.0019	2	1	0	0.0	1	0	0	0	-	0	0	-	0
viii-Lbor	0.0019	3	3	1	7.5	0	0	3.5	0	-	0	0	-	0
ii-vii	0.0010	1	1	0	5.0	0	-	-4.0	0	-	0	0	-	0
vii-xiii	0.0284	10	8	0	21.0	0	-	3.0	2	0	3.0	0	-	0
vii-x	0.0031	3	2	0	6.0	1	-	-5.0	0	-	0	0	-	0
x-xiv	0.0410	16	8	0	28.5	6	-	7.5	1	0	1.0	1	0	1.0
x-xv	0.0402	19	13	0	38.0	2	-	2.5	3	0	2.5	1	-	1.0

Branches are between nodes as numbered in Figure 2. 'CI1' is the frequency of the highly consistent characters (CI = 1) on a branch in the simultaneous analysis tree. A '-' represents a node not recovered in the independent analyses. 'HS' = hidden synapomorphy index [14] and 'PB' = partition Bremer support.

### Character dynamics: data interaction

Spearman's correlation analysis revealed a significantly positive relationship between RAG2 and the other data partitions (ρ^2 ^= 0.432, P = 0.003) but failed to find evidence for interaction between cyt *b*, Actin, and control region partitions. However, the distribution of positive and negative partitioned Bremer support values within the simultaneous analysis topology suggested localized character conflict among data partitions (Figure 3). The greatest amount of dispersion in partitioned Bremer support was between the control region and other data partitions at the shortest nodes in the simultaneous analysis tree (Table 2), where the partitioned Bremer support for the control region were consistently negative (Figure 3). In addition, the hidden synapomorphy values for the control region were significantly negative (sign test, P = 0.007), with a frequent displacement of synapomorphies with the addition of data in the simultaneous analysis (Table 2). The implications of these findings are two-fold: (1) the control region was responsible for the majority of data conflict, and (2) the relatively weak support for some nodes was a result of this conflict. In contrast, cyt *b *did not demonstrate a significant pattern of gain or loss of synapomorphies (P = 0.13) due to simultaneous analysis. In fact, cyt *b *contributed an additional synapomorphy to 6 different nodes following simultaneous analysis (Table 2). The number of synapomorphies contributed by the nuclear DNA data partitions did not change between independent and simultaneous analyses (data not shown), which indicated they were not a source of character conflict.

### Character conflict exploration

**Figure 5:**
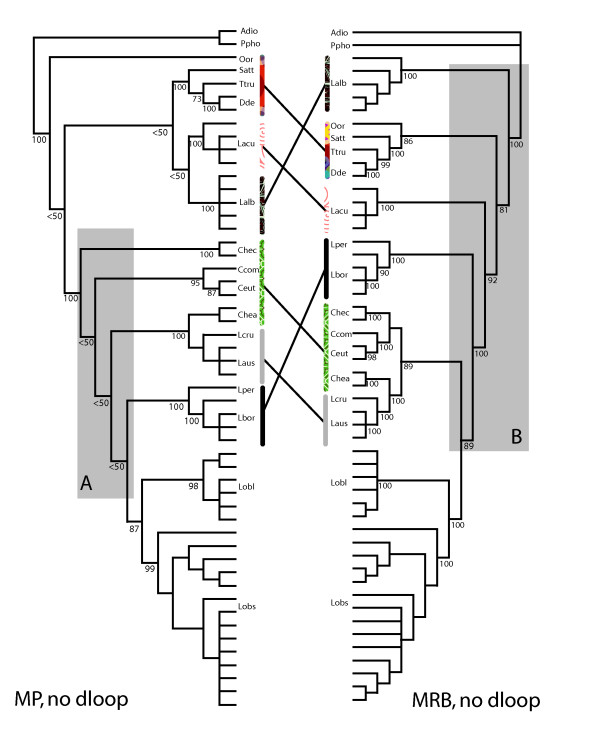
**Comparison of results from phylogenetic analyses without control region data**. Maximum parsimony (left) and Bayesian (right) topologies are presented with bootstrap proportions and posterior probabilities (respectively) at each node. Patterned bars demarcate topological differences between the two phylogenies, and gray boxes highlight the regions of the trees where the majority of topological incongruence occurs. Note in each tree the lack of monophyly of *Cephalorhynchus*, the polyphyly of *Lagenorhynchus*, and the change in relationships among genera in the Lissodelphininae indicated by linked vertical bars.

We explored further the effects of character conflict on the topology by excluding the control region and performing a parsimony and Bayesian analysis with the same parameters as the simultaneous analysis. The topologies from the truncated parsimony and Bayesian analyses were markedly different from each other and from the simultaneous analysis cladogram, and failed in both cases to recover the monophyly of key genera of the Lissodelphininae (Figure 5). In particular, neither analysis produced a monophyletic *Cephalorhynchus*, which has been well supported in other phylogenetic studies [22]. These results indicated that the control region data contributed to the overall resolution of the simultaneous analysis tree, particularly within the Lissodelphininae, despite evidence for character conflict, and that this was true whether or not the data were analyzed with parsimony or Bayeisan methods. In addition, the removal of the control region data did not produce a monophyletic *Lagenorhynchus*, which indicated character conflict alone was not responsible for the polyphyly of this genus.

## Discussion and conclusion

### Lissodelphininae and *Lagenorhynchus *systematics

Overall, our study demonstrates that the simultaneous analysis of nuclear and mitochondrial process partitions, coupled with better taxonomic sampling, provided greater phylogenetic resolution than cyt *b *alone [13,14]. The simultaneous parsimony and Bayesian analyses increased measures of support for 7 nodes, and resolved relationships among 5 clades that were previously ambiguous in the analysis of LeDuc et al. [13] (Figure 2). Furthermore, our study recovered a monophyletic *Cephalorhynchus*, and increased posterior probabilities for 4 nodes (i.e., iv, vi, viii, xvii, and xviii Figure 2) compared to the Bayesian tree of May-Callado and Agnarsson [14]. These results are consistent with other studies that suggest phylogenetic analysis based on mitochondrial data alone can be misleading, and that single gene trees are not always accurate representations of species trees. Even in cases where there was apparent conflict among characters, our data suggest synergistic interaction in the simultaneous analysis, and that *a priori *exclusion of data because of potential conflict may inhibit the recovery of robust phylogenetic hypotheses. For example, the removal of the control region, the putative source of character conflict, produced spurious results with inconsistencies among and within topologies from parsimony and Bayesian analyses (Figures 2 and 5), and these inconsistencies were not trivial. In both the parsimony and Bayesian analysis without the control region, well-supported monophyletic groups were rendered poly- or para-phyletic (e.g., *Cephalorhynchus*), and deeper-level relationships were extensively altered (Figures 2 and 5). The reliability of Bayesian posterior probabilities as a measure of node support has been recently called into question. [23-26]. Therefore the increased nodal support derived from Bayesian analyses should be interpreted in light of this controversy, and although bootstrap values and posterior probabilities are not directly comparable, a conservative approach might be to consider them as lower and upper bounds of node reliability [23]. Nevertheless, our results support previous studies that suggest data sets interact when analyzed simultaneously [16,17,20,27,28], and that properties of data emerge that are not detectable when partitions are analyzed separately [15,16,20,27,28].

### Character dynamics

Several methods have been proposed to quantify congruence and conflict in simultaneous analyses [15,17,20,29,30]. Although these measures are useful descriptors of inconsistency among analyses, many do not provide a quantitative measure with which to compare trees derived from independent data partitions. It has been argued (see recent review by Grant and Kluge [21] and references therein) that an *a posteriori *analysis of various character partitions against a simultaneous analysis tree is part of a heuristic approach for evaluating the utility of different partitions to resolve relationships among lineages that potentially differ in evolutionary rates and/or overall level of divergence. In this study, we have attempted through a suite of statistical tests (Table 3) to quantitate *a posteriori *the degree of interaction and phylogenetic utility of data partitions in combined analyses. In many of these tests we chose to use a subset of parsimony informative characters that lacked homoplasy (CI = 1) (Table 3). The logic behind this approach is that in any given topology those characters with a CI of 1 are less likely to be displaced from a node with the addition of future data (i.e., have phylogenetic "inertia") than less consistent characters, and thus represent a conservative class of characters by which to evaluate the utility of data partitions. We acknowledge that even grossly erroneous trees can contain some characters with a CI of 1, and all tree topologies are subject to change with the addition of data – this is true of any working hypothesis. However, we argue that the *a posteriori *evaluation of the behavior of CI = 1 characters provides one means to quantitatively evaluate the nature of data interaction and utility, and thus identify potential sources of character incompatibility.

**Table 3:** Summary of statistical tests used to measure *a posteriori *the utility and interaction of data partitions.

Tests	Question(s)	H_0_	Statistical test
Utility of each data partition to tree resolution	Does the strength of the signal decay for each partition as branch lengths increase?	No relationship between frequency of CI1 characters and branch length	Linear regression
	Is there variation in the contribution of data partitions to node support relative to branch length?	Equal frequency of CI1 characters for each data partition across branch lengths	Chi-square
	Is there variation in relative support a partition contributes to short and long branches?	Uniform proportion of CI1 for each data partition across branch lengths	Kruskal-Walis
Utility of 3^rd ^codon positions	Are third positions less informative than other codon positions?	Uniform frequency of CI1 characters among codon positions	Chi-square
Conflict among data partitions in the simultaneous analysis	Is there evidence for interaction among data partitions?	Even distribution of PBS across node heights	Spearman's rank correlation
	Does simultaneous analysis displace synapomorphies?	No displacement of synapomorphies (HS ≤ 0) in combined analysis	Sign test

CI1 = characters with consistency index of 1 (CI = 1); HS = hidden synapomorphy index; PBS = partitioned Bremer support.

For example, our results indicate there is a significant, positive relationship between CI = 1 characters and branch length for cyt *b*, dloop, and Actin (Figure 4), which is not unexpected given that longer branches are generally expected to have greater numbers of character changes than shorter branches. We also expected that the dloop, all things being equal, would have greater number of CI = 1 characters than cyt *b *or Actin since the dloop evolves more rapidly than other mitochondrial and nuclear DNA regions in cetaceans (and mammals in general). In contrast, we observe that both the frequency of CI = 1 characters and the strength of the CI = 1 frequency/branch length relationship is greater in cyt *b *than the dloop and Actin partitions (Table 2, Figure 4). One interpretation of these results is that the evolution of the dloop is so rapid that the phylogenetic information is reduced by multiple substitutions. However, if this were the case, we would expect deterioration of phylogenetic signal with increased branch length – we did not observe this pattern. Instead, we suggest an alternative interpretation – that these results indicate something about the phylogenetic utility and/or the degree of conflict of the dloop with other characters in combined analysis. For example, we have evidence that the dloop is a source of character conflict, and the deviation of the expected results from the linear regression analysis may be an additional indicator of this conflict. Yet, despite this conflict, the significantly positive relationships between branch lengths and CI = 1 characters suggest that all data partitions (to varying degrees) contribute to tree resolution, even in the presence of character conflict. Therefore, this method is a general indicator of the relative ability of each data partition to resolve relationships among OTU's with varying degrees of evolutionary divergence, and provides an example of the emergent properties of characters in combined analysis.

Our statistical tests suggest that the frequency and distribution of CI = 1 characters was not uniform for any data partition, suggesting that the rate of evolution was not constant among lineages (Figure 4). This is not surprising given that heterotachy and covariation are common phenomena in molecular data. However, we were interested to find that, although the frequency of CI = 1 characters varied along lineages, the relative proportion of CI = 1 characters contributed by each data partition was not statistically different (Figure 4). Our interpretation of this finding is that the short branches at the base of the Lissodelphininae were the result of an actual acceleration in rate of evolution along these lineages and not an artefact of the pattern of evolution of any given data partition. In this regard, we suggest this, or similar, statistical tests might be useful in identifying regions in a tree that represent rapid divergence, which are often difficult to resolve with phylogenetic analysis due to the relatively fewer number of informative characters along very short branches. The implications of these results are two-fold. First, the consistent lack of uniformity among data partitions in the number of highly consistent characters provides strong evidence for shifts in the rate of evolution of delphinid lineages since they shared a common ancestor with the Phocoenidae approximately 10 million years ago. Second, the rapid reduction in the number of highly consistent characters in the middle of the simultaneous analysis tree, and the corresponding short branch lengths and large number of lineages indicates that a shift in diversification rate occurred most recently at the base of the subfamily Lissodelphininae (Figure 4). Given that 8 of the 10 species in this monophyletic subfamily are found only in the Southern Hemisphere, this may represent a rapid anti-tropical divergence of lineages south of the equator following equatorial transgression from the north.

### Taxonomic implications

Our results support the monophyly of the Lissodelphininae (node iii, Figure 2), and therefore concur with Le Duc et al. [13] and May-Callado and Agnarsson [14] that the genus *Lagenorhynchus *is polyphyletic. Le Duc et al. [13] proposed that the generic name *Lagenorhynchus *remain with *L. albirostris*, the type specimen of the genus [7], and that the genus *Leucopleurus *[10] should be resurrected for *L. acutus *according to taxonomic precedence. In contrast to Le Duc et al. [13], the parsimony and Bayesian analyses in this study recovered the monophyly of *L. acutus *and *L. albirostris *(Figure 2, node x), and supports a close relationship of these species with the *Delphinus/Stenella/Tursiops *clade, although the Bayesian and parsimony analysis did not agree on the relationships among these clades (Figure 2). We therefore propose that both *L. acutus *and *L. albirostris *retain their current generic designation until further analyses are performed with better taxonomic sampling outside of the Lissodelphininae.

Second, the addition of nuclear and mitochondrial process partitions provided strong support for the paraphyly of *Lagenorhynchus *within the subfamily Lissodelphininae (Figure 2). In addition, both parsimony and Bayesian analyses support the sister-group relationship of *L. obliquidens *and *L. obscurus *and the monophyly of the *Cepharlorhynchus*, which has been proposed by other molecular phylogenetic studies [12,13,22,32]. This study provides improved phylogenetic resolution for other groups including the monophyly *L. cruciger *and *L. australis*, and the placement of this clade within the Lissodelphininae (Figure 2). Our evidence concurs with the results of Le Duc et al. [13] that *L. cruciger*/*L. australis *are monophyletic and should be placed into a separate genus, *Sagmatius*, first described from *Sagmatius amblodon *[11] and later synonymized with *L. australis *[33]. The monophyletic *L. obliquidens *and *L. obscurus *are more problematic and will require the description of a new genus.

Molecular data have helped to resolve some of the problems associated with dolphin taxonomy, but it is apparent that issues related to variation in the rate of evolution within the family continue to make the resolution of some relationships problematic. The next step in this study would be to combine morphology and DNA sequence characters in a simultaneous analysis. For example, Fraser and Purves [34] found distinct differences among genera of dolphins based on a suite of characters in the sinuses related to the structure and function hearing. A re-evaluation of these characters and additional molecular loci for all members of the Delphinidae might provide resolution of evolutionary relationships for problematic taxa.

## Methods

### Specimens

Tissues were obtained from biopsy punches and skin swabs from living animals and post-mortem samples from beach-cast or net-caught individuals (Table 1). Skin swabs were collected following the non-invasive procedure of Harlin et al. [35], preserved in either 90% ETOH or a solution of 20% dimethylsulfoxide (DMSO) saturated with salt, and stored at -20°C. When possible, DNA from the same individuals was used to amplify and sequence all genes. In some cases, pre-existing sequences from GenBank were used to complete the data matrix (Table 1).

**Table 1:** Species, sample sizes, and sources of genetic materials used in this study.

Taxon (abbreviation)	Cytb	D-loop	Actin	RAG2
	n	n	n	n
*Lagenorhynchus obscurus (Lobs)*	14^a^	14^a^	14^a,r^	14^a^
*Lagenorhynchus obliquidens (Lobl)*	6^b^	6^b^	6^q^	6^b^
*Lagenorhynchus cruciger (Lcru)*	1^h^	1^l^	-	1^g^
*Lagenorhynchus australis (Laus)*	3^a,i^	3^a^	3^a^	3^a^
*Lagenorhynchus albirostris (Lalb)*	5^s,c^	5^s,c^	5^s,c^	5^s,c^
*Lagenorhynchus acutus (Lacu)*	4^d^	4^d^	4^d^	4^d^
*Cephalorhynchus hectori (Chec)*	2^j,g^	2^m^	2^g^	2^g^
*Cephalorhynchus commersonii (Ccom)*	1^t^	1^t^	1^t^	1^t^
*Cephalorhynchus eutropia (Ceut)*	2^k^	2^n^	-	-
*Cephalorhynchus heavisidii (Chea)*	2^e^	2^e^	2^e^	2^e^
*Lissodelphis peronii (Lper)*	1^u^	1^u^	-	1^u^
*Lissodelphis borealis (Lbor)*	4^v^	4^v^	4^v^	4^v^
*Delphinus delphis (Dde)*	2^c^	2^c^	2^c^	2^c^
*Stenella attenuata (Satt)*	1^c^	1^c^	1^c^	1^c^
*Tursiops truncatus (Ttru)*	1^c^	1^c^	1^c^	1^c^
*Australophocoena dioptrica (Adio)*	1^f^	1^f^	1^f^	1^f^
*Phocoena phocena (Ppho)*	1^f^	1^o^	1^f^	1^f^
*Orcinus orca (Oor)*	1^f^	1^p^	1^f^	1^f^
TOTAL	52	52	48	50

**a**. A. Harlin; **c**. Texas A&M Cooperative Wildlife Collection; **d**. R. L. Honeycutt; **e**. M. Meyer, Sea Fisheries Research Institute, Capetown, South Africa; **f**. C. S. Baker, University of Auckland, New Zealand; **g**. F. Pichler, University of Auckland, New Zealand. GenBank Accessions: **h**. AF084068; **i**. AF084069; **j**. AF084071; **k**. AF084072, U13128; **l**. AF084072; **m**. AF057997, AF057998; **n**. AF393555, AF393553; **o**. U09694; **p**. M60409; **q**. AF140826–AF140831; **r**. AF140832–AF140834; **w**. AY011968. SWFSC National Marine Mammal Tissue Bank, La Jolla, California: **b**. Z15004, Z15860, Z25424, Z25409–Z25411; **s**. Z17311, Z17318, Z17319, Z23522; **t**. Z40; **u**. Z6996; **v**. Z23163, Z25407, Z25408, Z26303

Fifty-two individuals from eight genera and 18 species in the family Delphinidae were examined, with emphasis on representation of the genus *Lagenorhynchus *and other members of the subfamily Lissodelphininae (Table 1). All 12 species of the putative subfamily and members of the genus *Lagenorhynchus *(i.e., *Lagenorhynchus *sp., *Cephalorhynchus *sp., and *Lissodelphis *sp.) are represented (Table 1). In addition, members of the *Tursiops*/*Delphinus*/*Stenella *clade identified by LeDuc et al. [13], and the killer whale (*Orcinus orca*), a taxon thought to be the basal delphinid lineage [36], were included. *Australophocoena dioptrica *and *Phocoena phocoena *from the family Phocoenidae were selected as outgroup taxa because of their sister-taxon relationship to the Delphinidae [37,38].

### DNA isolation and amplification

Total genomic DNA was isolated using either a standard phenol-chloroform protocol [39] or a Qiagen DNeasy kit (Qiagen, Valencia, California). The polymerase chain reaction (PCR) was used to amplify fragments of four genes including: (1) the complete mitochondrial cytochrome b (cyt *b*) gene (1040 nucleotides), (2) 474 base pairs (bp) of the mitochondrial control region, (3) 995 bp of nuclear DNA (nDNA) intron I of the muscle Actin gene, and (4) 474 bp of the coding region of the nDNA recombination activating gene 2 (RAG2). External primer sets included: (1) cyt *b *– 766F (5'-gaaaaaccaycgttgtwattcaact-3') and 766R (5'-gtttaattagaatytyagctttggg-3'); (2) control region – tRNA-Pro and Dlp5 of Baker et al. [40]; (3) Actin – *Lagenorhynchus*-specific LagAct1 (5'-gatttggtccctctatgtctct-3' and LagAct2 – 5'-tacttttgaacttgccacctac-3'). Actin primers were designed from published cetacean sequences [41] and used to amplify the majority of ingroup taxa. Act1 [41] and Act1385H (5'-cttgtgaactgattacagtcc-3') (Palumbi, unpublished) were used to amplify fragments for outgroup taxa and others that failed to amplify with the *Lagenorhynchus*-specific primers. RAG2 primers were the same as those reported by Murphy et al. [42]. PCR conditions were generally consistent across loci with adjustments made to annealing temperatures. Approximately 1–2 μl of DNA template were included in 50 μl PCR reactions containing the following: 5 μl each of 10× Amplitaq PCR buffer (Perkin Elmer, Boston, Massachusetts), MgCl (25 mM), and deoxynucleotide triphosphates (dNTP's,10 mM), and 1 μl each of bovine serum albumin (BSA, 10 mg/ml), each primer (10 μM), and 1 μl Amplitaq (Perkin Elmer, Boston, Massachusetts) DNA polymerase (5 U/μl). Thermocycler conditions were 94°C for 2 min followed by 35 cycles at 92°C for 30 s, annealing 30 s, and extension at 72°C for 30 s. Published annealing temperatures were used with the following exceptions: 765F/766R, 50°C; LagActin1/2, 58°C; Act1/1385H, 56°C. Amplicons were electrophoresed in 1.5% agarose-TBE (tris, boric acid, EDTA) and visualized under UV light, and prior to sequencing, excess oligonucleotides and dNTP's were removed with either Qiagen (Qiagen, Valencia, California) spin-columns or an Exonuclease I-Shrimp Alkaline Phosphotase (Exo-Sap) enzymatic procedure. Approximately 2 ng of cleaned PCR product per 100 bp of amplicon length was sequenced using ABI BigDye (Applied Biosystems, Foster City, California) cycle sequencing chemistry and an ABI 377 automated sequencer. All amplicons were sequenced in both directions. Internal primer pairs for cyt *b *(560, 5'-gcaaccctaacacgattcttcg-3'; 610, 5'-ccagtttcgtgtaggaataatagg-3') and Actin (Act5-L, 5'-ccactactttaggcag-3'; M13Act5R-H, 5'-tgtaaaacgacggccagtctgcctaaactagtgg-3' (S. Palumbi, unpublished) were used in sequencing reactions to obtain complete overlap in both directions. Sequences generated in this study are accessioned in the NCBI GenBank database: (1) Dloop: EF092925–EF092969; (2) Cytb: EF093009–EF093055; (3) Actin: EF092970–EF093008; (4) RAG2: EF093056–EF093105.

### Sequence alignment and heterozygosity

Sequenced fragments were edited and compiled with the program Sequencher v. 4.1 (Gene Codes Corp., Ann Arbor, Michigan). A consensus of sense and anti-sense strands for each individual and data partition were compiled and exported to MacClade vs. 4.05 [43]. Sequences of the four data partitions were concatenated into a single string of nucleotide characters for the same individual when possible, or a combination of fragments from members of the same species. Cyt *b *and RAG2 contained no length variable regions, thus alignment of these fragments was trivial. Amino acid translations of the open reading frames of RAG2 and cyt *b *were examined for stop codons to verify sequence orthology. Actin fragments contained minor genus-specific insertions and deletions that were revealed by alignment in Clustal X [44] with default parameters. The control region had one (CT)_n _length variable region of approximately 21 bp that was eliminated from analyses as primary homology could not be reliably assessed. There were an additional 29 sites with indels in the control region, 20 of which were present only in outgroup taxa (*A. dioptrica, P. phocoena*). Actin had 24 indel sites in three blocks (4, 8, and 12 sites) that were apomorphic (taxon-specific). All species were represented by fragments from the four data partitions with the exceptions: (1) *C. eutropia*, which lacked Actin and RAG2 fragments because we were not able to obtain tissue samples for this species, and (2) *L. cruciger *and *L. peronii *which lacked Actin fragments (Table 1).

All nucleotide ambiguities in Actin and RAG2 that resulted from two different, but equally strong, peaks on electropherograms were considered as evidence for potential heterozygous sites. All such positions were assigned IUPAC ambiguity codes, and considered as ambiguous characters in subsequent phylogenetic analyses. All potential heterozygous sites were species-specific and occurred in only a single individual of each species. PCR reactions consistently produced only one Actin or RAG2 amplicon, which were subjected to a BLAST search to verify sequence identity. In all BLAST searches, amplicons retrieved sequences from either delphinid or mammalian taxa as the closest match, providing further evidence for successful amplification of target loci.

### Phylogenetic analyses

Cladograms were generated with the program PAUP* v.4.0b10 [55] under the optimality criterion of parsimony, with all characters equally weighted. Previous studies suggest that gaps can be phylogenetically informative in both coding and non-coding regions [45,46], thus all gaps were considered as 5th states in parsimony analyses. The number of taxa and characters precluded the use of exhaustive search options. Therefore, we performed a heuristic search with 1000 random additions of taxa, 100 trees held at each replicate, and tree-bisection-reconnection (TBR) branch-swapping. In addition, phylogenetic analyses were performed separately for mitochondrial (cyt *b *and control region) and nuclear (Actin and RAG2) data sets with the same heuristic search parameters. Clade support for the simultaneous analysis tree was evaluated with bootstrap [56] and decay indices [57]. The bootstrap procedure was replicated 10,000 times, each as full heuristic search with random addition of sequences and TBR branch swapping with 100 trees held at each step. Decay indices for the simultaneous analysis tree were derived from a heuristic search of constraint trees created with the program TreeRot v.2b [58]. Heuristic search parameters were the same for the original simultaneous analysis.

There is well-documented variation in the rate of evolution within and among mitochondrial and nuclear genomes; therefore, a Bayesian analysis was performed with the program Mr. Bayes v. 3.0 [59] in order to compare phylogenetic hypotheses derived from parsimony and Bayesian methods. Each data partition was assigned an independent (unlinked) general-time-reversal (GTR) model of substitution with a proportion of invariant sites and variation in substitution rates among sites. Two independent runs of 2 million iterations were performed, each with four chains, three hot, one cold, sampling one tree in 10. Adequate mixing and convergence of chains was examined visually with the program Tracer v.1.1.1 [60]; posterior probabilities for each clade were derived from trees sampled after the burn in period.

The Bayesian method does not have an option for gaps to be coded as 5^th ^character states, and thus they are treated as missing data in our Bayesian analysis. Some studies have used a separate gap matrix to incorporate indels into Bayesian analyses (e.g., [47]), and recently there have been advances that allow the explicit treatment of gaps in the simultaneous derivation of sequence alignment and tree topology within parsimony, Bayesian, and likelihood analytical frameworks [48-54]. Such methods are effective particularly in dealing with regions difficult to align (e.g., length variable regions). Since our data set contains relatively few indels, the majority of which are not phylogenetically informative (see above), we opted not to further explore our data with these methods. Such topics deserve thorough treatment beyond the scope of this study.

### Character dynamics: phylogenetic utility

The behavior of combining characters from different process partitions was evaluated by examining the relative contribution, or utility, of data partitions to resolving relationships within the simultaneous analysis tree. Branch support indices were partitioned (i.e., "partitioned Bremer support") with the method of Baker and DeSalle [16] to measure the relative contribution of each data set to node support. The larger the partitioned Bremer support is for a given partition at a particular node, the greater the relative contribution of that partition to the support of that node [16]. We were also interested in the relative phylogenetic utility of nuclear and mitochondrial data per sequenced nucleotide. Therefore, we subsequently standardized the partitioned Bremer values for each node by the number of nucleotides sequenced.

A number of methods have been proposed to quantify *a posteriori *the contribution of a particular data partition to topological support in a simultaneous analysis [15]. These methods generally include a measure of the relative number of unambiguous character changes attributable to a given data partition [15, 30, 61, 62]. In this study, we chose to quantify at each node in the simultaneous analysis tree a subset of unambiguous character changes that have a consistency index (CI) of 1, that is, they change to one of the four possible states (A, G, C, or T) once only in the tree at a single node. Because these characters lack homoplasy in a given tree, we suggest that these characters are less likely to be affected by data interaction than characters that display homoplasy, and thus are a phylogenetically conservative, and potentially useful, set of characters for *a posteriori *evaluation of characters.

We use CI = 1 characters to examine two aspects of data partition utility (Table 3). First, we wished to determine if the amount of phylogenetic signal in each data partition decreases over time (i.e., with level of divergence). The idea is that if the number of highly consistent CI = 1 characters that a data partition contributes to a node is constant over time (i.e., there is no decay in phylogenetic signal with increased divergence), we would expect a positive, linear relationship between the number of CI = 1 characters and branch length. This would indicate that a data partition is well suited to address hypotheses of evolutionary relationships at the levels of divergence present in this study. Second, we were interested in whether or not the relative contribution of each data partition to node resolution was consistent among branches of variable lengths (Table 3). Consistent patterns of evolution over time (i.e., the relative frequency and rate of substitutions at particular nodes) among multiple loci can provide evidence that variation in branch lengths among regions of a topology represent the actual evolutionary history of a lineage. In this manner it would be possible to determine if short branches represent periods of rapid or simultaneous taxonomic divergence (i.e., are "hard" polytomies), or are a consequence of some aspect of the data, e.g., incompatibility, or a rate of evolution ill suited to resolve short branches.

To test these hypotheses, we first estimated the number of CI = 1 characters per branch by labelling all unambiguous character changes on the simultaneous analysis and independent analysis trees with their consistency index in MacClade v. 4.01 [43]. Branch lengths (all inter-node distances) were estimated in PAUP* v.4.0b10 [55] by holding the simultaneous analysis tree topology constant and optimizing character state evolution along branches via maximum likelihood with empirical base frequencies, a general-time-reversible (GTR) model, and a gamma shape parameter estimated from the data (n = 8 categories). These estimates of branch lengths and the number of CI = 1 characters per branch were used in subsequent testing of hypotheses regarding phylogenetic utility. To address the question of phylogenetic signal over time, a linear regression was used to examine the relationship between branch length and number of CI = 1 characters along each branch (Table 3). To address the second question of node resolution, we performed two tests (Table 3). First, a chi-square test for homogeneity of proportions was used to determine if CI = 1 characters were evenly distributed within the simultaneous analysis tree with respect to branch length. Second, a Kruskal-Walis non-parametric statistic for multiple samples was used to test if the proportion of CI = 1 characters contributed by each data partition was uniform across branches of different lengths. We pooled data into 4 branch length categories (0–0.0019; 0.0020–0.0049; 0.0050–0.019; 0.0200–0.0500) to increase sample sizes for these tests

Additionally, the relative contribution of each partition to a node was examined by calculating the consistency index of unambiguous character changes at first, second, and third codon positions in the simultaneous analysis tree. A chi-square was used test the null hypothesis of homogeneity of highly consistent characters among codon positions. All statistical tests were performed with SPSS v. 11 statistical package. Null hypotheses were rejected if P ≤ 0.05.

### Character dynamics: data interaction

A Spearman's rank correlation of partitioned Bremer support values was used to determine the nature and significance of interaction among data partitions in the simultaneous analysis topology. Partitioned Bremer values for data partitions at a particular node can be used as indicators of conflict among data partitions – positive values indicate support and negative values suggest conflict. A Spearman's rank correlation of partitioned Bremer support values from the simultaneous analysis tree [63] was performed to measure the level and magnitude of character interaction on a node-by-node basis. A significantly positive correlation coefficient is evidence that partitions support different nodes in similar proportions; a negative correlation indicates significant conflict among data partitions. A non-significant correlation suggests that topological support is not associated with any combined pairs of data partitions [17]. We also calculated the "hidden synapomorphy" [15] index, defined as the difference in the number of unambiguous character changes at nodes in simultaneous and separate analyses. The number of unambiguous changes for each node was determined with MacClade v.4.01 [43]. A negative hidden synapomorphy index indicated the displacement, or loss, of unambiguous synapomorphies from a node as a result of combining data, whereas positive values suggested synergistic data interaction. The greater the magnitude of the hidden synapomorphy value, the greater the interaction among characters at that node. A sign test, a non-parametric analog of the t-test, was used to determine if the simultaneous analysis had a significant effect on the frequency of synapomorphy displacement.

## Authors' contributions

ADHC designed the study, collected tissue samples, carried out the laboratory work, performed all phylogenetic and statistical analyses, and drafted the manuscript. RLH assisted with the study design and multiple drafts of the manuscript. All authors have read and approved the final manuscript.
